# Probiotic Enhanced Intestinal Immunity in Broilers against Subclinical Necrotic Enteritis

**DOI:** 10.3389/fimmu.2017.01592

**Published:** 2017-11-20

**Authors:** Hesong Wang, Xueqin Ni, Xiaodan Qing, Lei Liu, Jing Lai, Abdul Khalique, Guangyao Li, Kangcheng Pan, Bo Jing, Dong Zeng

**Affiliations:** ^1^Animal Microecology Institute, College of Veterinary Medicine, Sichuan Agricultural University, Chengdu, China; ^2^Ya’an Agricultural Science and Technology Development Co., Ltd., Ya’an, China

**Keywords:** subclinical necrotic enteritis, *Lactobacillus johnsonii*, intestinal immunity, probiotic, broiler

## Abstract

Along with banning of antibiotics, necrotic enteritis (NE), especially subclinical NE (SNE) whereby no clinical signs are present in chicks, has become one of the most threatening problems in poultry industry. Therefore, increasing attention has been focused on research and application of effective probiotic strains, as an alternative to antibiotics, to prevent SNE in broilers. In the present study, we evaluated the effects of *Lactobacillus johnsonii* BS15 on the prevention of SNE in broilers. Specifically, assessment determined the growth performance and indexes related to intestinal mucosal immunity in the ileum and cecal tonsil of broilers. A total of 300 1-day-old Cobb 500 chicks were randomly distributed into the following 5 groups: control group (fed with basal diet + de Man, Rogosa, and Sharpe liquid medium [normal diet]), SNE group (normal diet), BS15 group (basal diet + 1 × 10^6^ colony-forming units BS15/g as fed [BS15 diet]), treatment group (normal diet [days 1–28] + BS15 diet [days 29–42]), and prevention group (BS15 diet [days 1–28] + normal diet [days 29–42]) throughout a 42-day experimental period. SNE infection was treated for all chicks in the SNE, BS15, treatment, and prevention groups. The present results demonstrated that BS15 supplementation of feeds in BS15 and prevention groups exerted a positive effect on preventing negative influences on growth performance; these negative influences included low body weight gain and increased feed conversion ratio caused by SNE. Although no changes were detected in all determined indexes in cecal tonsils, BS15-treated broilers were free from SNE-caused damage in villi in the ileum. BS15 inhibited SNE-caused decrease in immunoglobulins in the ileum. In the lamina propria of ileum, T cell subsets of lymphocytes influenced by SNE were also controlled by BS15. BS15 affected antioxidant abilities of the ileum and controlled SNE-induced mitochondrion-mediated apoptosis by positively changing contents and/or mRNA expression levels of apoptosis-related proteins. These findings indicate that BS15 supplementation may prevent SNE-affected growth decline mainly through enhancing intestinal immunity in broilers.

## Introduction

Necrotic enteritis (NE), a poultry disease that is mainly caused by *Clostridium perfringens* (CP), was first reported in 1961 and remains important in the poultry industry to date (1). Over the recent decades, NE has been prevented and treated by supplementation of antibiotics in feed (2). However, along with banning of antibiotics, NE has become one of the most threatening problems in the poultry industry. Subclinical NE (SNE) especially presents poor performance without mortality. As chicks show no clinical sign of the disease, most economic losses result from the presence of subclinical forms (3, 4). Aggravating the condition, meat containing affected nutrients or toxins may be purchased and consumed by customers (5). Therefore, an effective alternative to antibiotics must be developed for the prevention of SNE in broilers. Aside from the application of polysaccharides (6) and vaccines (7), probiotics is becoming a common method for prevention of SNE in postantibiotic era (8, 9). However, difficulty arises from determining the mechanism by which probiotics affect the gastrointestinal tract because probiotic strains exert their beneficial effects *via* different mechanisms in which other microbiota may be involved (10, 11). Kang et al. (12) reported that although live probiotics or their metabolites were found to interact with diverse immune cells and thus play a role as immune modulators, the effects of probiotics in prevention or modulation of diseases diversify even within the same species. In addition, Jacobsen et al. (13) previously demonstrated the difficulty of reliably extrapolating *in vitro* influences of probiotics to *in vivo* conditions. Therefore, although numerous studies have focused on the positive effects of probiotics in animals, the mechanisms by which probiotics can successfully exert beneficial effects on promoting growth performance and preventing diseases remain unclear (14).

*Lactobacillus johnsonii* BS15 (CCTCC M2013663), a probiotic strain which was proven to prevent non-alcoholic fatty liver disease in obese mice, was isolated from homemade yogurt collected from Hongyuan Prairie, Aba Autonomous Prefecture, China (15). In our recent previous studies, we applied BS15 to broilers and observed that BS15 supplementation can improve growth performance and significantly enhance the nutritional value of meat (16). Only live BS15 (not disrupted cells) may exert health benefits mainly by improving lipid metabolism, intestinal development, and gut microflora in the small intestine at the starter phase (17). In our preliminary study on the prevention of SNE, BS15 controlled growth performance, lipid deposits, and fatty acid composition of chicken meat during subclinical CP infection (5). Thus, on the basis of all our recent studies, we evaluated the effect of dietary supplementation of BS15 on prevention of SNE in broilers. Aiming to determine the relationship between preventive effects of BS15 and intestinal immunity, the present study assessed growth performance and indexes related to intestinal immunity in the ileum and cecal tonsil of broilers.

## Materials and Methods

### Feed Preparation

Viable counts of BS15 cell preparations were evaluated by heterotrophic plate counts after maintaining cultures in de Man, Rogosa, and Sharpe (MRS) broth at 37°C for 36 h under an anaerobic environment. Supplementation was performed before each feeding. The basic procedure was as follows: approximately 10 mL of BS15 solution/MRS liquid medium [diluted with the same amount of phosphate-buffered saline (PBS)] was thoroughly mixed with 1,000 g of diet (17).

### Animals and Treatment

**Figure 1:**
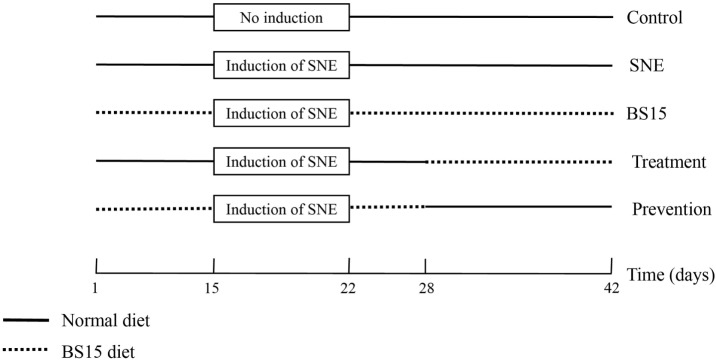
Schematic outline of the experimental design. SNE, subclinical necrotic enteritis.

A total of 300 1-day-old male chicks (Cobb 500) with similar body weights were purchased from Chia Tai broiler hatchery (Chengdu, China). Chicks were weighed and divided into five treatment groups. Each group consisted of 6 replicates with 10 birds per replicate at the Key Laboratory of Animal Disease and Human Health of Sichuan Province, Sichuan Agricultural University. Birds were fed *ad libitum* and provided with free access to water throughout the entire experiment. Artificial light was provided all day using fluorescent light. Room temperature was maintained at 33°C for the first 3 days and then gradually reduced by 3°C weekly until a temperature of 24°C was reached; the final temperature was maintained until the end of experiment. Table 1 shows starter and finisher diet formulas. All diets were formulated to satisfy or exceed the *National Research Council* requirements for broilers (18). Five bird groups were fed with diets in mash form as follows: control group (basal diet + MRS liquid medium [normal diet]), SNE group (normal diet), BS15 group (basal diet + 1 × 10^6^ colony-forming units [cfu]; BS15/g as fed [BS15 diet]), treatment group (normal diet [days 1–28] + BS15 diet [days 29–42]), and prevention group (BS15 diet [days 1–28] + normal diet [days 29–42]). Each feed amount was consumed by chicks within 3 h and resupplied every 3 h. All chicks in SNE, BS15, treatment, and prevention groups were gavaged orally with 20,000 *Eimeria acervulina* oocysts and 5,000 *Eimeria maxima* oocysts per bird at 15 days of age and then with 1 mL of CP (2.2 × 10^8^ cfu/mL) from days 18–22 for SNE induction. The control group was gavaged with 1 mL of PBS at 15 days of age and days 18–22 (4, 19). Figure 1 summarizes treatments of each group. Chicks were weighed, and feed intake was recorded in the morning at days 7, 14, 21, 28, 35, and 42. Average daily gain (ADG), average daily feed intake (ADFI), and feed conversion ratio (FCR) were also calculated. All animal experiments were performed in accordance with the guidelines for the care and use of laboratory animals and approved by the Institutional Animal Care and Use Committee of Sichuan Agricultural University (approval number: SYXKchuan2014-187).

**Table 1:** Composition of basal diets for broiler chickens.

Ingredient^a^	Starter diet (%) 1–21 days	Finisher diet (%) 22–42 days
Ground yellow corn	56.00	59.50
Soybean meal	37.00	32.90
Soybean oil	3.66	4.70
Ground limestone	0.57	0.50
Dicalcium phosphate	1.80	1.60
Salt	0.30	0.30
Choline chloride	0.10	0.10
dl-Met	0.24	0.12
Micronutrients^b^	0.33	0.33
Calculated nutrients level		
ME (MJ/kg)	12.40	12.80
*Clostridium perfringens*	21.20	19.70
Lys	1.19	1.08
Met	0.50	0.40
Met + Cys	0.86	0.74
Ca	0.85	0.77
Nonphytate P	0.44	0.40

*^a^Ingredient and nutrient composition are reported on an as-fed basis*.

*^b^Micronutrients were provided per kilogram of diet: vitamin A (all-trans retinol acetate), 12,500 IU; cholecalciferol, 2,500 IU; vitamin E (all-rac-a-tocopherol acetate), 18.75 IU; vitamin K (menadione Na bisulfate), 5.0 mg; thiamine (thiamine mononitrate), 2.5 mg; riboflavin, 7.5 mg; vitamin B6, 5.0 mg; vitamin B12, 0.0025 mg; pantothenate, 15 mg; niacin, 50 mg; folic acid, 1.25 mg; biotin, 0.12 mg; Cu (CuSO_4_⋅5H_2_O), 10 mg; Mn (MnSO_4_⋅H_2_O), 100 mg; Zn (ZnSO_4_⋅7H_2_O), 100 mg; Fe (FeSO_4_⋅7H_2_O), 100 mg; I (KI), 0.4 mg; and Se (Na_2_SeO_3_), 0.2 mg*.

### Sampling

On the morning of day 28, six birds (one bird per cage) from the control, SNE, and BS15 groups were randomly selected and sacrificed by exsanguination under anesthesia in accordance with institutional animal care guidelines. Subsequently, samples from the ileum and cecal tonsil were removed and washed with ice-cold sterilized saline. These samples were then frozen in liquid nitrogen and stored at −80°C for subsequent analysis of antioxidant abilities, enzyme activities, and/or gene expression. Total ileal RNA was extracted using RNAiso Plus (TaKaRa, Dalian, China) in accordance with manufacturer’s guidelines. Quality and quantity of isolated RNA were assessed from the ratio of absorbance values at 260 and 280 nm and by agarose gel electrophoresis. First-strand complementary DNA (cDNA) was synthesized from 1 µg of total RNA using PrimeScriptTM RT reagent kit with gDNA Eraser (TaKaRa, Dalian, China). All cDNA products were frozen at −20°C until further use.

### Detection by Flow Cytometry Method (FCM)

Another six birds from the same three groups were selected and sacrificed. Ileum and cecal tonsil were collected from each bird. After isolating intraepithelial lymphocytes (IELs) and lamina propria lymphocytes (LPLs) from the ileum, IELs and LPLs from ileum and cecal tonsil were subjected to FCM to determine CD3^+^, CD3^+^CD4^+^, and CD3^+^CD8^+^ T cell percentages as described by Wu et al. (20). The methods described by Todd et al. (21) and Reséndiz-Albor et al. (22) were performed to isolate IELs and LPLs of ileums, respectively.

### Histological Observation and Immunohistochemistry

Another six birds from the control, SNE, and BS15 groups were selected and sacrificed by the previously mentioned method. Samples of ileum sections (1 cm^2^) and cecal tonsils were collected and fixed in 10% neutral-buffered formalin, processed and trimmed, and embedded in paraffin. Sections with 5-µm thickness were stained with hematoxylin and eosin and observed by light microscopy. A light microscope (Olympus, Japan) was used to observe morphological changes. Immunoglobulin (Ig) A^+^ B cells were localized in the ileum, as observed by immunohistochemistry. Immunohistochemical staining and counting were performed as described by Liu et al. (23). Staining was performed in three independent replicates to confirm results. Tissue sections were dewaxed in xylene, rehydrated through a graded series of ethanol, washed in distilled water and PBS, and then blocked for endogenous peroxidase by incubation with 3% H_2_O_2_ in methanol for 15 min. Sections were subjected to antigen retrieval by microwaving in 0.01 M sodium citrate buffer (pH 6.0). Additional washing in PBS was performed before incubation in 10% normal goat serum at 37°C for 30 min. Tissue sections were incubated with diluted (1:100) primary antibodies at 4°C overnight. Antibodies used included polyclonal mouse anti-chicken IgA heavy chains (SouthernBiotech 8330-01, Birmingham, AL, USA). For negative controls, tissue sections received PBS in place of the primary antibody. After washing in PBS, sections were exposed to 1% biotinylated secondary antibody goat anti-mouse IgG (ZSGB-BIO SP Kit, ZSGB-BIO, Beijing, China) at 37°C for 1 h. Slices were then incubated with horseradish peroxidase-streptavidin (ZSGB-BIO SP Kit) for 30 min at 37°C. To visualize immunoreaction, tissue sections were immersed in diaminobenzidine hydrochloride. Reactions were monitored microscopically and stopped by immersion in distilled water as soon as a brown staining has been visualized. Slices were lightly counterstained with hematoxylin, dehydrated in ethanol, cleared in xylene, and mounted on glass slides. IgA^+^ B cells were counted using a computer-supported imaging system connected to a light microscope (Olympus AX70, Tokyo, Japan) and then quantified using Image-Pro Plus 5.1 (Silver Spring, MD, USA) image analysis software. Six sections were measured in each group, with each section measured five times and averaged.

### Immunoglobulins and Antioxidant Indexes

Levels of Igs, including IgG, IgM, IgA, and secretory IgA (sIgA), in the ileum and cecal tonsil were quantified using chicken-specific enzyme-linked immunosorbent assay (ELISA) kits (RD Ltd., USA) following manufacturer’s instructions. Antioxidant indexes in ileum were measured using commercial kits by Nanjing Jiancheng Bioengineering Institute (Nanjing, Jiangsu, China); these indexes included total antioxidation capacity (T-AOC), activities of catalase (CAT), superoxide dismutase (SOD), glutathione peroxidase (GSH-Px), inhibition of hydroxy radical (IHR), and malondialdehyde (MDA) and GSH contents.

### Apoptosis-Related Proteins, Cytokines, Matrix Metalloproteinase-2 (MMP-2), and Nrf-2

Levels of apoptosis-related proteins, including Bcl-2, Bax, and caspase-3, and cytokines, including interleukin (IL)-2, IL-6, IL-8, IL-10, IL-17, tumor necrosis factor-alpha (TNF-α), and interferon-gamma (IFN-γ), in the ileum were quantified using ELISA kits specific for chicken (RD Ltd., USA). mRNA expression levels were determined according to our recent study (17). By using the prepared cDNA products from the ileum and cecal tonsil, polymerase chain reaction (PCR) was performed using a CFX96 Real-Time PCR Detection System (Bio-Rad, Hercules, CA, USA) with SYBR Premix Ex Taq™ PCR kit (TaKaRa, Dalian, China). Thermocycling protocol was as follows: 5 min at 95°C, followed by 40 cycles of 15 s denaturation at 95°C, and 30 s annealing/extension at optimum temperature (Table 2). A final melting curve analysis was used to monitor purity of PCR products. Table 2 presents primer sequences for targeted genes. Standard curves were obtained from serial dilution of samples. _ΔΔ_Ct method was used to estimate mRNA abundance. Ct is calculated as (Ct_target_ − Ct_GAPDH_)_treatment_ − (Ct_target_ − Ct_GAPDH_)_control_, where glyceraldehyde 3-phosphate dehydrogenase is the eukaryotic housekeeping gene used to normalize relative gene expression levels. All samples (*n* = 6) in each group were analyzed in triplicate. Mean values of measurements were used to calculate mRNA expression levels of Bcl-2, Bax, caspase-3, cytochrome *c* (Cytc *c*), apoptotic protease activating factor 1 (Apaf-1), caspase-9, IL-2, IL-6, IL-8, IL-10, IL-17, TNF-α, IFN-γ, MMP-2, and Nrf-2 in the ileum.

**Table 2:** Primer information on genes for RT-qPCR.

Gene name	Primer sequence (5 → 3)	Tm (°C)/size (bp)	Accession
*IL-2*	F: TCTGGGACCACTGTATGCTCT R: ACACCAGTGGGAAACAGTATCA	56.0	AF000631
*IL-6*	F: CAAGGTGACGGAGGAGGAC R: TGGCGAGGAGGGATTTCT	56.0	AJ309540
*IL-8*	F: GGCTTGCTAGGGGAAATGA R: AGCTGACTCTGACTAGGAAACTGT	55.9	NM_205498.1
*IL-10*	F: CGGGAGCTGAGGTGAA R: GTGAAGAAGCGGTGACAGC	55.0	AJ621614
*IL-17*	F: CTCCGATCCCTTATTCTCCTC R: AAGCGGTTGTGGTCCTCAT	55.0	AJ493595
*IFN-*γ	F: AGCTGACGGTGGACCTATTATT R: GGCTTTGCGCTGGATTC	58.0	Y07922
*TNF-*α *(LITAF)*	F: TGTGTATGTGCAGCAACCCGTAGT R: GGCATTGCAATTTGGACAGAAGT	55.5	AY765397
*Cytc c*	F: CAGTGCAACTTCAACCATACCA R: AACCAGCCAGTCCACAACAA	61.0	NM001079478
*Apaf-1*	F: AAGGGCATAAGGAAGCAATCAA R: CAGCACAAGAAAGAACAGCACC	61.0	XM416167
*Bax*	F: TCCTCATCGCCATGCTCAT R: CCTTGGTCTGGAAGCAGAAGA	61.9	XM422067
*Bcl-2*	F: GATGACCGAGTACCTGAACC R: CAGGAGAAATCGAACAAAGGC	62.0	NM205339
*Caspase-3*	F: TGGCCCTCTTGAACTGAAAG R: TCCACTGTCTGCTTCAATACC	62.0	NM204725
*Caspase-9*	F: CGAAGGAGCAAGCACGACAG R: CCGCAGCCCTCATCTAGCAT	61.5	AY057940
*MMP-2*	F: AGCTGCACCGTCACCAATCAT R: CCTGCATCTGTGCAGCTGTTG	58.0	U07775.1
*Nrf-2*	F: ATCACGAGCCCTGAAACCAA R: GGCTGCAAAATGCTGGAAAA	61.0	D49365.1
*GADPH*	F: GGTGAAAGTCGGAGTCAACGG R: CGATGAAGGGATCATTGATGGC	58.4/108	NM204305

*TNF-α, tumor necrosis factor-alpha; IFN-γ, interferon-gamma; Cytc c, cytochrome c; Apaf-1, apoptotic protease-activating factor 1; MMP-2, matrix metalloproteinase-2; GADPH, glyceraldehyde-3-phosphate dehydrogenase; IL, interleukin*.

### Data Analysis

All results were expressed as mean ± SD and analyzed by one-way ANOVA. Duncan’s multiple range test was used for multiple comparison when a significant interaction was detected. All statistical analyses were conducted using SigmaPlot for Social Sciences version 12. Differences at *P* < 0.05 were considered statistically significant. Data on growth performance were obtained on a cage basis (*n* = 6), whereas other information were based on individual broilers (six replicates of one chick per cage).

## Results

### Growth Performance

**Figure 2:**
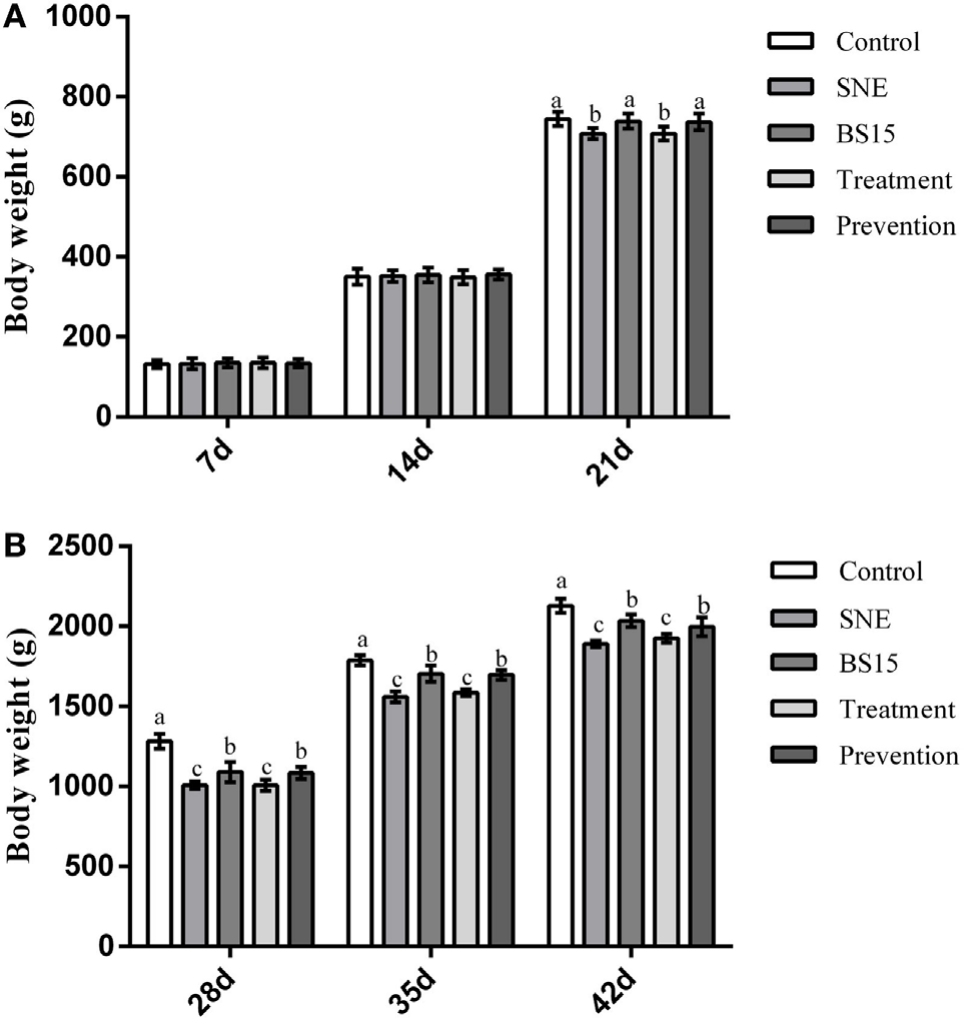
Body weight gain at 7, 14, 21, 28, 35, and 42 days. Bars with different letters significantly differ on the basis of Duncan’s multiple range tests (*P* < 0.05). Data are presented as means ± SD (*n* = 6). **(A)** At day 21, subclinical necrotic enteritis (SNE) and treatment groups showed lower body weight than the other three groups (*P* < 0.05). **(B)** Higher body weights were observed (*P* < 0.05) in the BS15 and prevention groups than in SNE and treatment groups, respectively, at 28, 35, and 42 days.

Table 3 and Figure 2 show growth performance results. At day 21, the SNE and treatment groups presented lower ADG and body weight than the other three groups (*P* < 0.05). Higher body weights were observed in the BS15 and prevention groups than in the SNE and treatment groups, respectively, at days 28, 35, and 42 (*P* < 0.05). High ADG and FCR were also observed in BS15 and prevention groups at days 22–42 (*P* < 0.05). As shown in Table 3, no significant differences were observed in days 1–21 and overall ADFI among all groups (*P* > 0.05), whereas a significantly lower ADFI was observed in the SNE group compared with the other four groups at days 22–42 (*P* < 0.05). At days 22–42, the control group showed significantly higher ADFI compared with the SNE and treatment groups (*P* < 0.05), whereas no significant differences were observed between BS15, treatment, and prevention groups (*P* > 0.05).

**Table 3:** *Lactobacillus johnsonii* BS15 on growth performance of SNE broilers.

Parameter	Control	SNE	BS15	Treatment	Prevention
**Days 1–21**					
ADFI (g/day)	61.16 ± 6.67	61.59 ± 4.54	62.12 ± 6.41	61.80 ± 7.21	61.97 ± 3.97
ADG (g/day)	33.45 ± 0.84^a^	31.72 ± 0.66^b^	33.20 ± 0.91^a^	31.70 ± 0.84^b^	33.12 ± 0.99^a^
FCR	1.83 ± 0.18^b^	1.94 ± 0.06^a^	1.87 ± 0.09^b^	1.95 ± 0.23^a^	1.88 ± 0.06^b^
**Days 22–42**					
ADFI (g/day)	122.35 ± 16.38^a^	108.17 ± 8.33^c^	117.97 ± 8.07^ab^	110.88 ± 11.47^bc^	115.32 ± 10.37^ab^
ADG (g/day)	65.92 ± 2.81^a^	54.91 ± 0.86^c^	62.25 ± 2.65^b^	56.60 ± 1.92^c^	60.39 ± 2.68^b^
FCR	1.86 ± 0.09^c^	1.97 ± 0.15^a^	1.90 ± 0.08^b^	1.96 ± 0.08^a^	1.91 ± 0.14^b^
**Overall (days 1–42)**					
ADFI (g/day)	91.75 ± 8.43	84.88 ± 5.14	90.04 ± 6.38	86.34 ± 6.00	88.64 ± 3.77
ADG (g/day)	49.69 ± 1.04^a^	43.32 ± 0.49^c^	47.72 ± 0.94^b^	44.15 ± 0.63^c^	46.75 ± 1.40^b^
FCR	1.85 ± 0.09^c^	1.96 ± 0.12^a^	1.89 ± 0.05^b^	1.96 ± 0.12^a^	1.90 ± 0.06^b^

*Data are presented as means ± SD (*n* = 6; cage was used as experimental unit). Means in the same row with no superscripts or with a common superscript letter do not differ significantly (*P* < 0.05)*.

*ADFI, average daily feed intake; ADG, average daily gain; FCR, feed conversion rate (ADFI/ADG); SNE, subclinical necrotic enteritis*.

### Histological Observation and Immunohistochemistry

**Figure 3:**
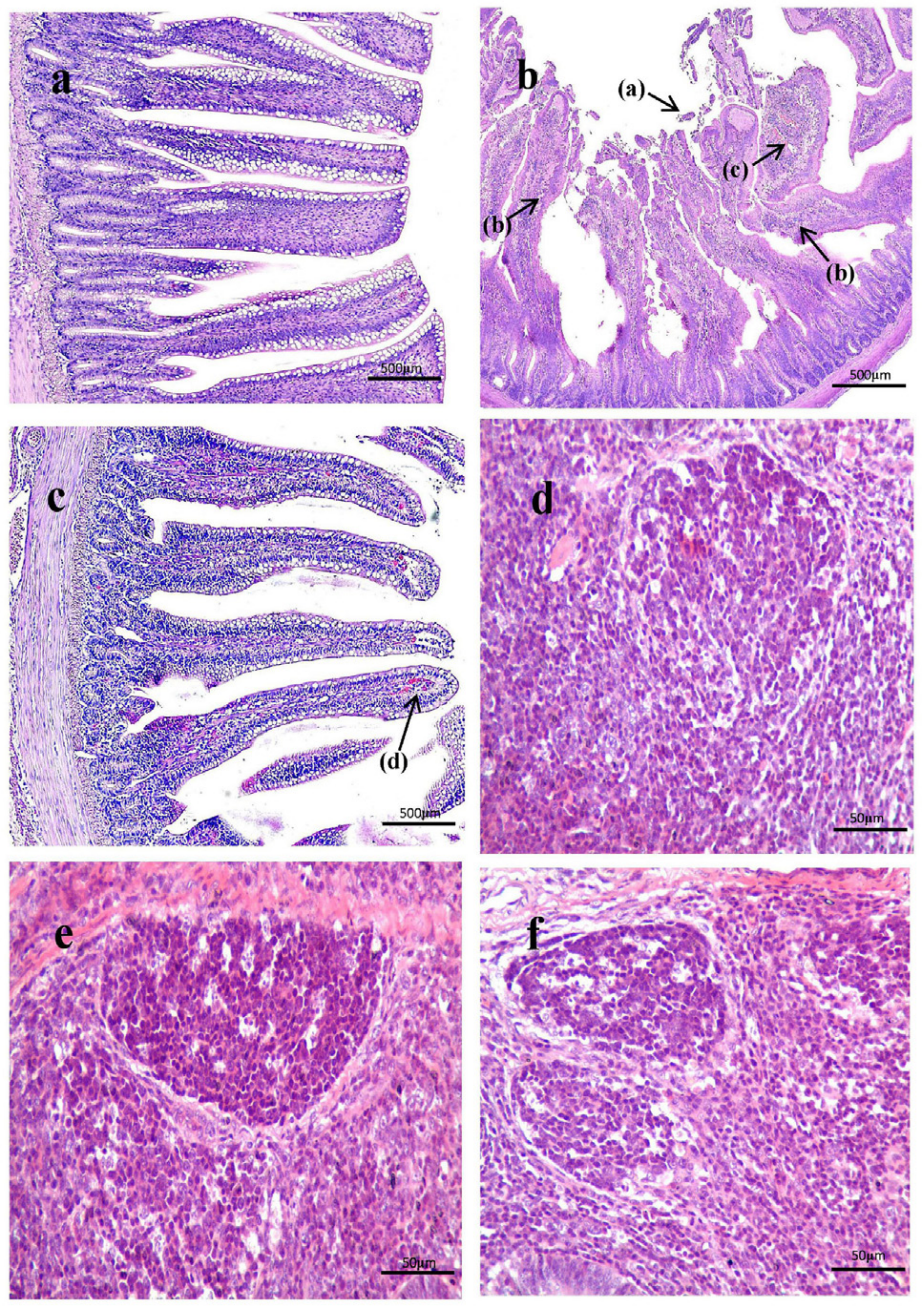
**(A–C)** Histological observation of ileum under original magnification (×40). **(A)** Ileum in the control group; **(B)** ileum in subclinical necrotic enteritis (SNE) group; **(C)** ileum in BS15 group; **(D–F)** histological observation of cecal tonsil under original magnification (×400); **(D)** cecal tonsil in the control group; **(E)** cecal tonsil in SNE group; **(F)** cecal tonsil in BS15 group. (1) Villi structure in the SNE group was destroyed and mainly presented the following features: shed epithelial cells (a), increased number of lymphocytes (b), and congested lamina propria (c). (2) In the BS15 group, lamina propriae were lightly congested (d). (3) No remarkable differences were observed in the cecal tonsil among the three groups.

**Figure 4:**
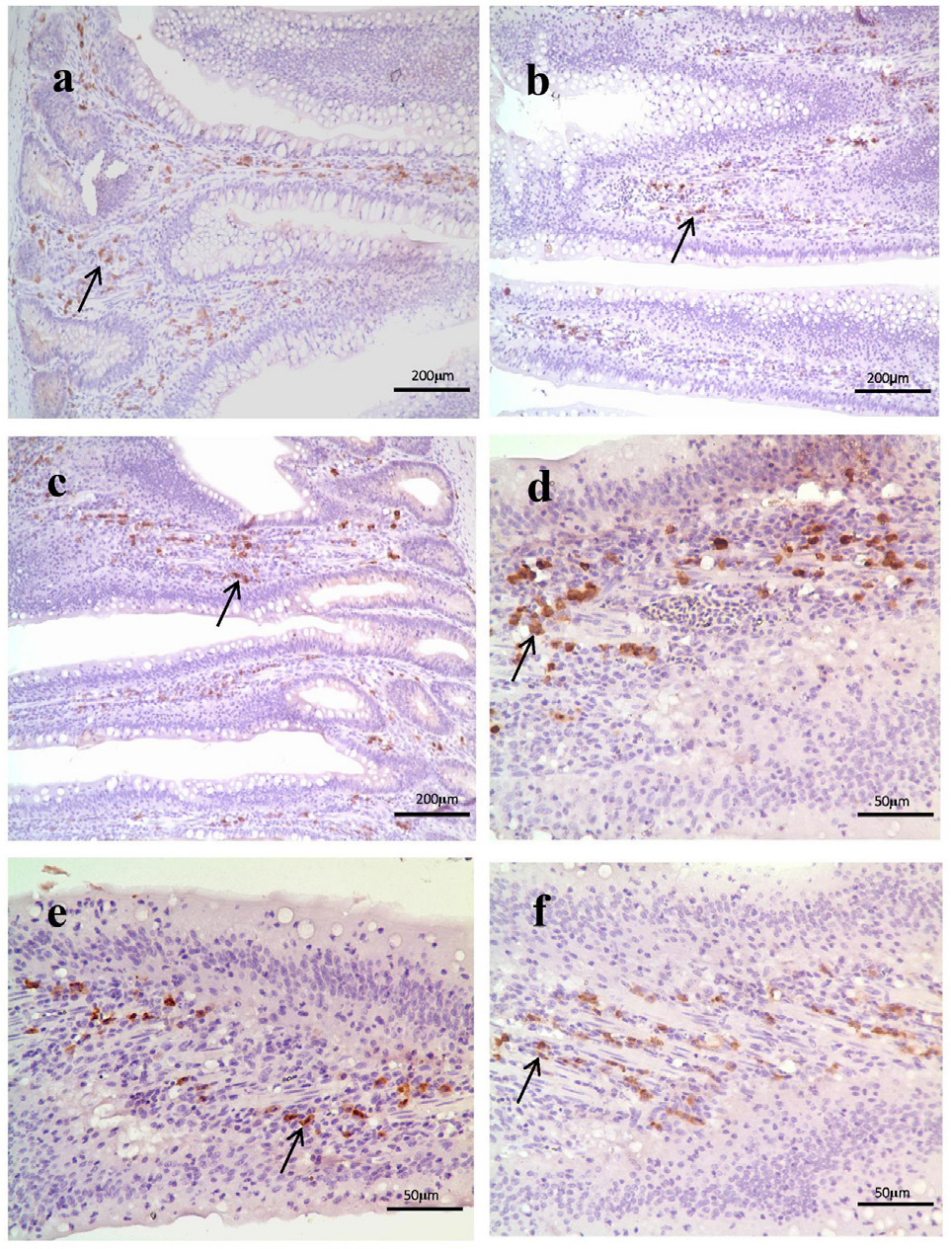
**(A–C)** IgA^+^ B cell numbers of the lamina propria in the ileum under original magnification (×100). **(A)** Ileum in the control group; **(B)** ileum in subclinical necrotic enteritis (SNE) group; **(C)** ileum in BS15 group; **(D–F)** IgA^+^ B cell numbers of the lamina propria in the ileum under original magnification (×400); **(D)** ileum in the control group; **(E)** ileum in SNE group; **(F)** ileum in BS15 group. In the BS15 group **(C,F)**, the number of IgA^+^ B cells in lamina propria of ileum was higher compared with that of the SNE group **(B,E)** but lower than that of the control group **(A,D)**.

**Figure 5:**
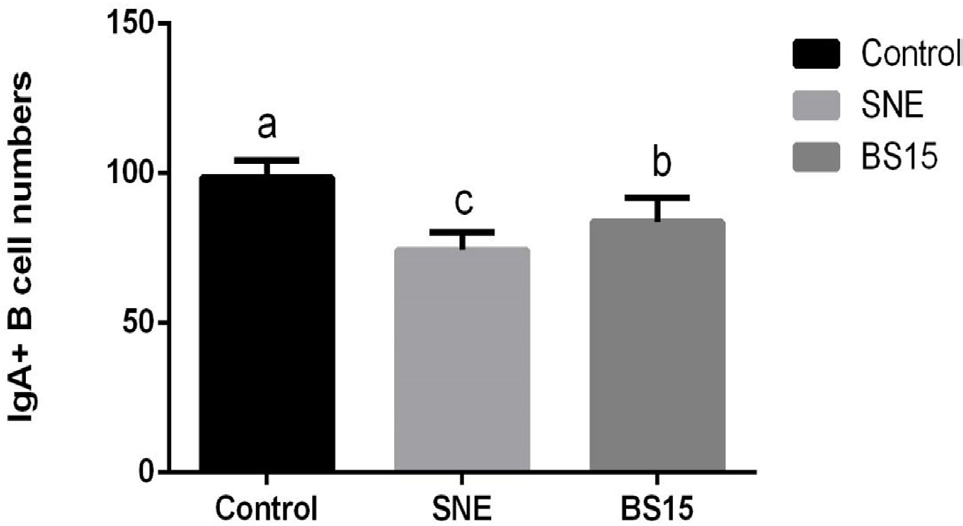
Changes in the numbers of IgA^+^ B cells in the lamina propria in the ileum. Bars with different letters significantly differ on the basis of Duncan’s multiple range tests (*P* < 0.05). Data are presented as mean ± SD (six replicates of one chick per cage). The number of IgA^+^ B cells in lamina propria of ileum in the BS15 group was higher compared with that of the subclinical necrotic enteritis (SNE) group (*P* < 0.05) but lower than that of the control group (*P* < 0.05).

As shown in Figure 3, histological examination showed intact villi structure in the control group, whereas those in the SNE group were destroyed and mainly presented the following features: shed epithelial cells, congested lamina propria, and increased number of lymphocytes. In the BS15 group, lamina propriae were lightly congested. No remarkable differences were observed in the cecal tonsil among the three groups. As shown in Figures 4 and 5, in the BS15 group, the number of IgA^+^ B cells in lamina propria of ileum was higher compared with that of the SNE group (*P* < 0.05) but lower than that of the control group (*P* < 0.05).

### Immunoglobulins

**Figure 6:**
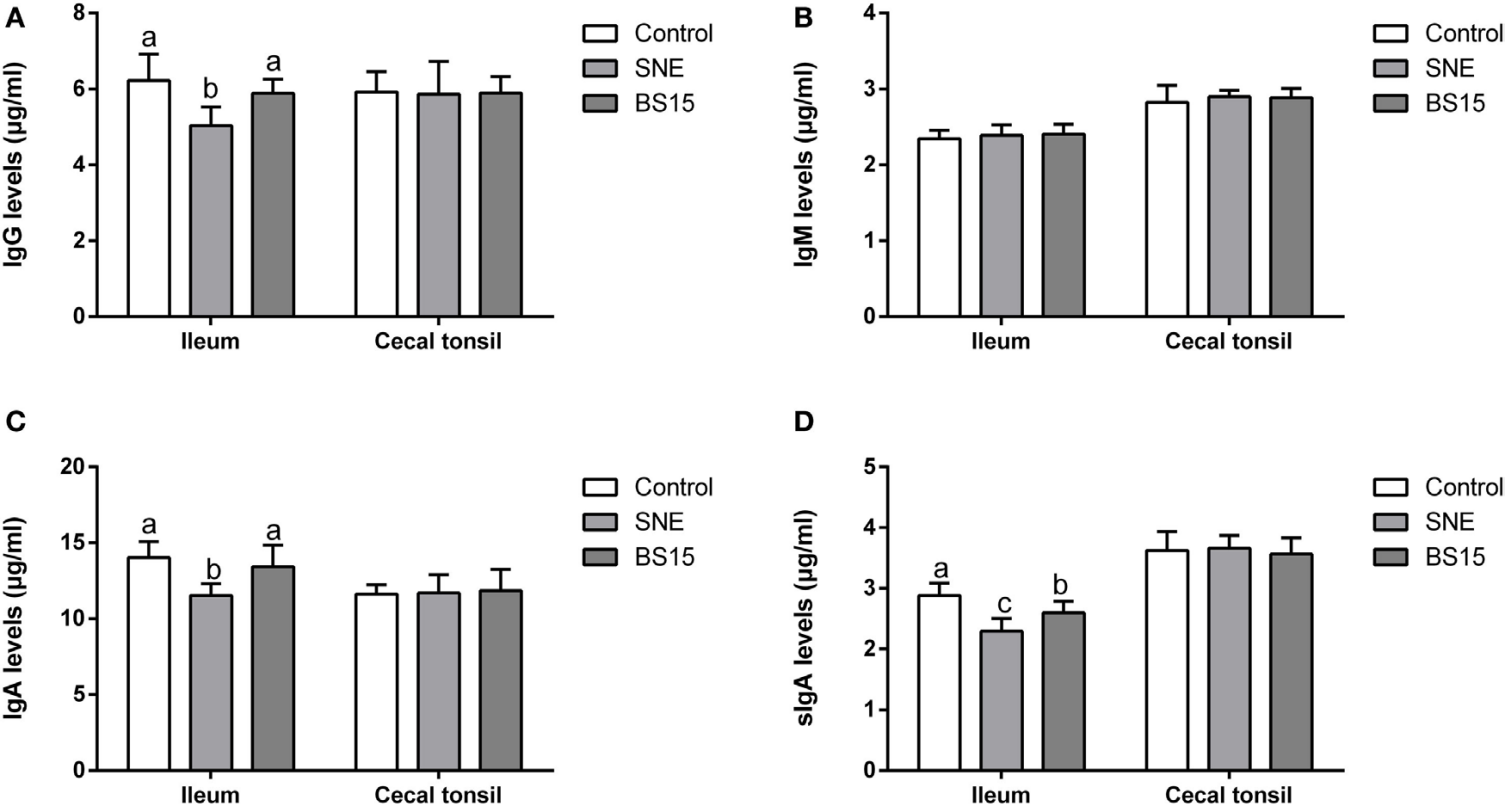
Changes in IgG, IgM, IgA, and sIgA levels in the ileum and cecal tonsil. Bars with different letters significantly differ on the basis of Duncan’s multiple range tests (*P* < 0.05). Data are presented as mean ± SD (six replicates of one chick per cage). **(A,C)** Levels of IgG and IgA in ileum were significantly lower in the subclinical necrotic enteritis (SNE) group than those in other groups (*P* < 0.05). **(A–D)** No significant difference was noted between BS15 and control groups (*P* > 0.05). **(D)** In the BS15 group, sIgA levels in the ileum were significantly lower than that of the control group (*P* < 0.05) and significantly higher than that of the SNE group (*P* < 0.05). **(B)** No difference was detected in IgM levels in the ileum among three experimental groups (*P* > 0.05). **(A–D)** No significant changes were observed on all determined Igs in the cecal tonsil (*P* > 0.05).

As shown in Figure 6, levels of IgG and IgA in ileum were significantly lower in SNE group than those in other groups (*P* < 0.05), but no significant difference was noted between BS15 and control groups (*P* > 0.05). In the BS15 group, sIgA level in the ileum was significantly lower than that of the control group (*P* < 0.05) and significantly higher than that in the SNE group (*P* < 0.05). No difference was detected in IgM levels in the ileum of the three experimental groups (*P* > 0.05), and no significant changes were observed in all determined Ig in the cecal tonsil (*P* > 0.05).

### T Cell Subsets

**Figure 7:**
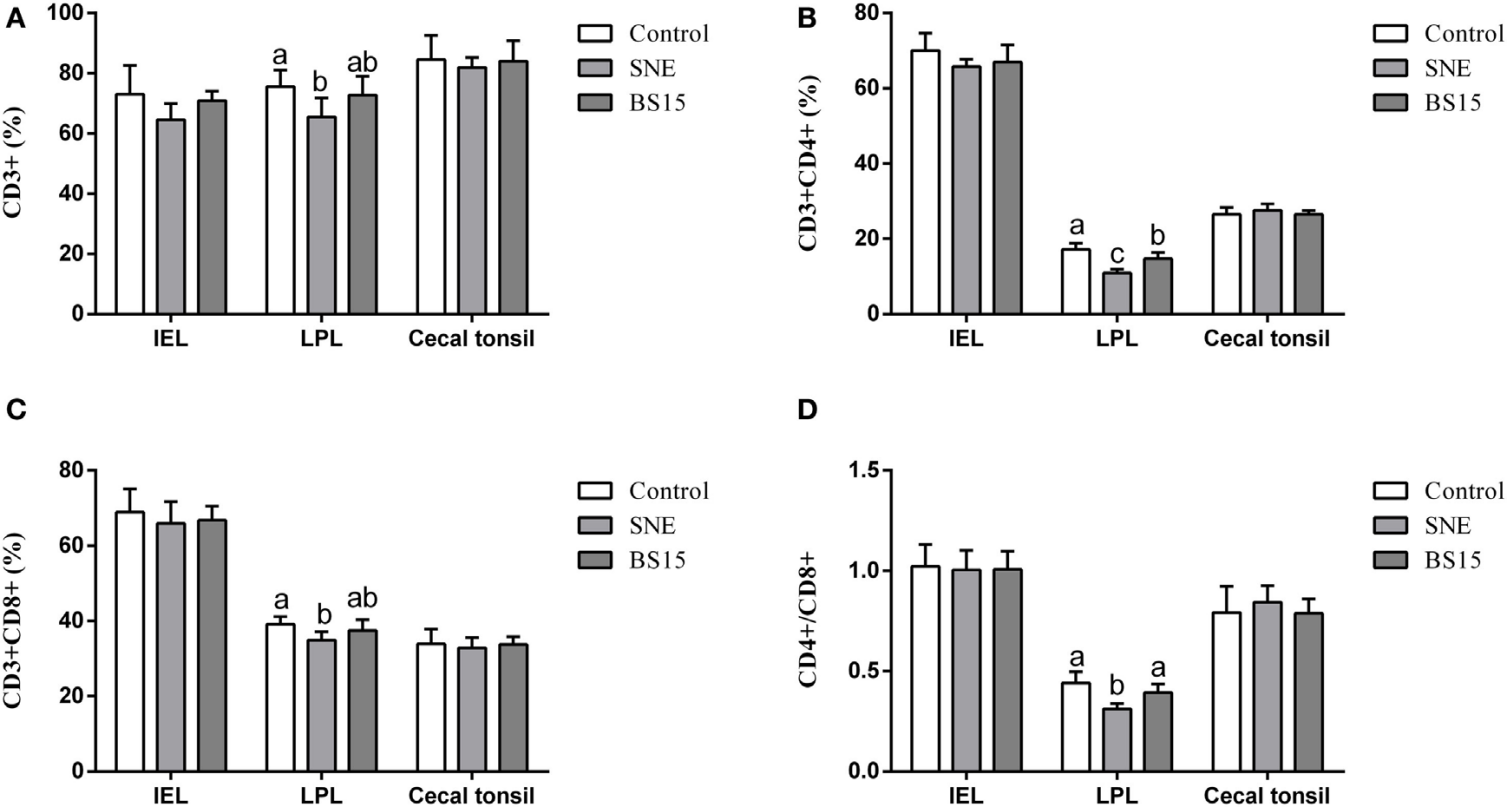
CD3^+^, CD4^+^, CD8^+^ T cells, and CD4^+^/CD8^+^ ratio in the ileum and cecal tonsil. Bars with different letters significantly differ on the basis of Duncan’s multiple range tests (*P* < 0.05). Data are presented as mean ± SD (six replicates of one chick per cage). **(A–D)** No significant changes were observed in intraepithelial lymphocytes (IELs) of ileum and cecal tonsil among the three experimental groups (*P* > 0.05). **(A–D)** In lamina propria lymphocytes (LPLs) of ileum, percentages of CD3^+^, CD4^+^, and CD8^+^ T cells and CD4^+^/CD8^+^ ratio were significantly lower in the BS15 group than those in the control group (*P* < 0.05). **(B,D)** Percentages of CD4^+^ T cells and CD4^+^/CD8^+^ ratio in BS15 group were higher than those in the SNE group (*P* < 0.05). **(A–D)** Except for CD4^+^ T cells, which were lower in number than those in the control group (*P* < 0.05), no significant difference was identified among the other three indexes (*P* > 0.05). **(A–D)** All determined indexes related to T cell subsets showed no difference in the cecal tonsil (*P* > 0.05).

Figure 7 displays results of T cell subsets as determined by FCM. No significant changes were observed in IELs of ileum and cecal tonsil among the three experimental groups (*P* > 0.05). In LPLs of ileum, percentages of CD3^+^, CD4^+^, and CD8^+^ T cells and CD4^+^/CD8^+^ ratio were significantly lower in BS15 group than those in the control group (*P* < 0.05), whereas percentages of CD4^+^ T cells and CD4^+^/CD8^+^ ratio in BS15 group were higher than those in the SNE group (*P* < 0.05). Except for CD4^+^ T cells, which were lower in number than those in the control group (*P* < 0.05), no significant difference was identified among the other three indexes (*P* > 0.05). All determined indexes related to T cell subsets showed no difference in cecal tonsil (*P* > 0.05).

### Antioxidant Indexes

**Figure 8:**
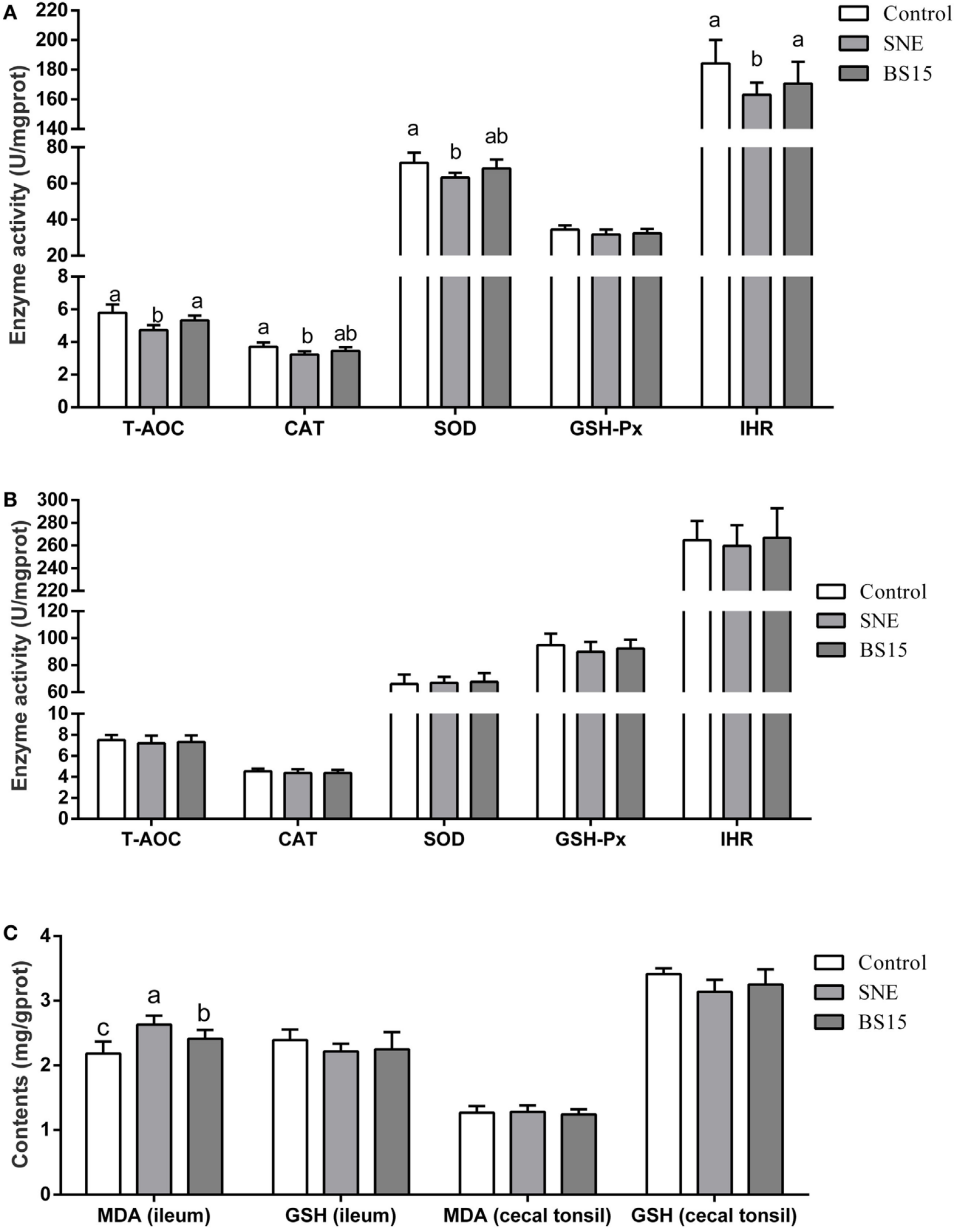
Antioxidant indexes in the ileum **(A,C)** and cecal tonsil **(B,C)**. Bars with different letters significantly differ on the basis of Duncan’s multiple range tests (*P* < 0.05). Data are presented as mean ± SD (six replicates of one chick per cage). T-AOC, total antioxidation capacity; CAT, catalase; SOD, superoxide dismutase; GSH-Px, glutathione peroxidase; IHR, inhibiting hydroxy radical; MDA, malondialdehyde; GSH, glutathione; SNE, subclinical necrotic enteritis. (1) T-AOC, CAT, SOD, and IHR activities in the ileum were significantly lower in the SNE group than in the control group (*P* < 0.05). (2) No significant differences (*P* > 0.05) in T-AOC and IHR in the ileum were observed between BS15 and control groups, but values for both were significantly higher than those in the SNE group (*P* < 0.05). (3) No significant change was observed in GSH-Px (*P* > 0.05). (4) MDA levels in the ileum of the BS15 group were lower than those of the SNE group (*P* < 0.05) and higher than that of the control group (*P* < 0.05). (5) GSH level remained unchanged based on the present results (*P* > 0.05). (6) All determined indexes in cecal tonsil showed no change (*P* > 0.05).

Figure 8 shows results on antioxidant indexes. As shown in Figures 8B,C, no changes were observed in all determined indexes in the cecal tonsil (*P* > 0.05). Results in Figure 8A show significantly lower T-AOC, CAT, SOD, and IHR activities in the ileum of the SNE group than those of the control group (*P* < 0.05). No significant differences (*P* > 0.05) in T-AOC and IHR in the ileum were observed between BS15 and control groups, but values for both were significantly higher than those in the SNE group (*P* < 0.05). No significant change was observed in GSH-Px (*P* > 0.05). As shown in Figure 8C, MDA levels in the ileum in BS15 group were lower than those in the SNE group (*P* < 0.05) and higher than those in the control group (*P* < 0.05). However, GSH level remained unchanged according to the present results (*P* > 0.05).

### Apoptosis-Related Proteins

**Figure 9:**
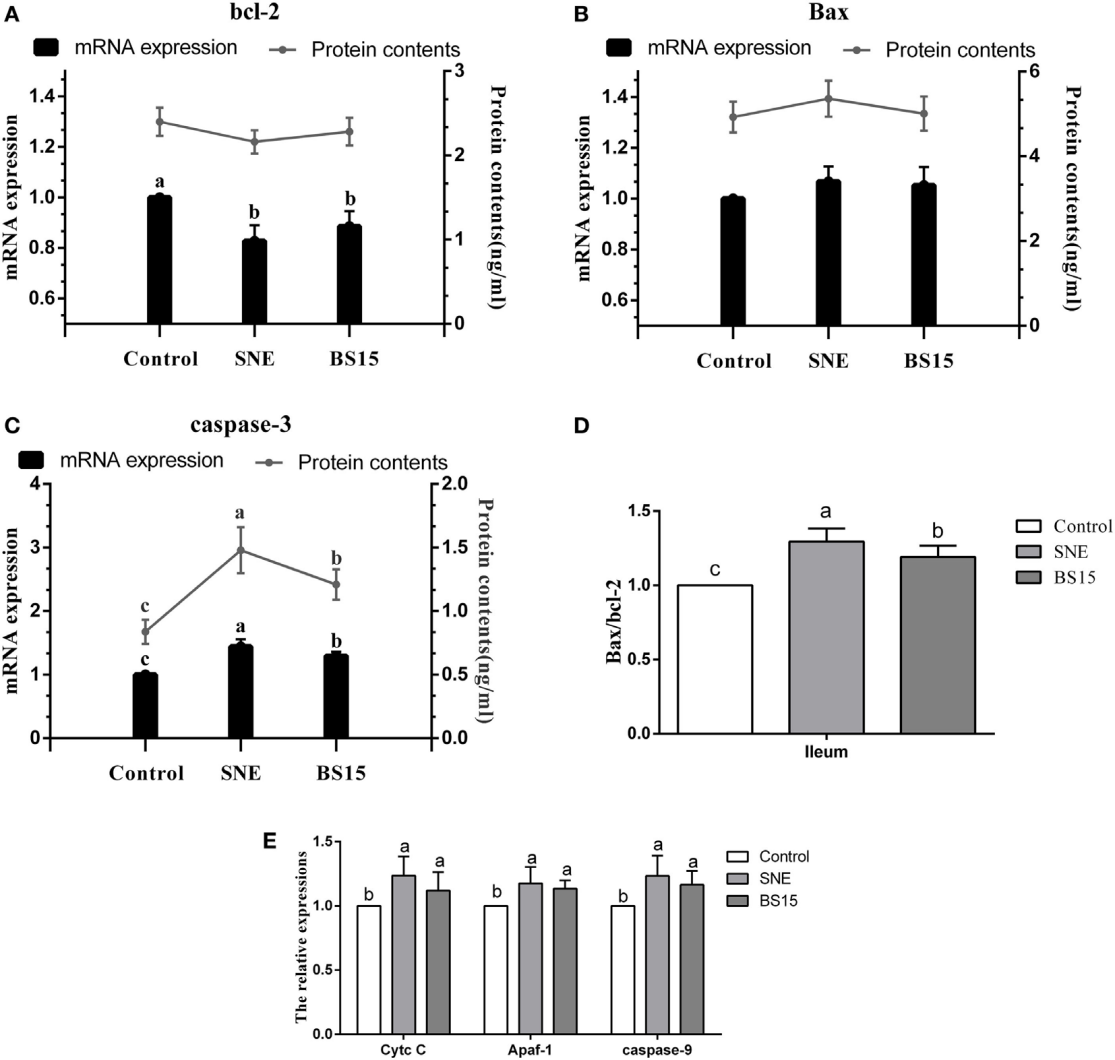
Levels of apoptosis-related proteins in the ileum. Bars with different letters significantly differ on the basis of Duncan’s multiple range tests (*P* < 0.05). Data are presented as mean ± SD (six replicates of one chick per cage). Cytc *c*, cytochrome *c*; Apaf-1, apoptotic protease activating factor 1. **(A)** Although no significant change in Bcl-2 protein content was detected (*P* > 0.05), mRNA expression levels of Bcl-2 in SNE and BS15 groups were significantly lower than those in the control group (*P* < 0.05). **(B)** Neither protein content nor mRNA expression level of Bax showed significant changes among the three groups (*P* > 0.05). **(C,D)** Both mRNA expression levels and protein contents of Caspase-3 and Bax/Bcl-2 ratio in the BS15 group were lower than those in the SNE group (*P* < 0.05) but higher than those in the control group (*P* < 0.05). **(E)** mRNA expression levels of Cytc *c*, Apaf-1, and Caspase-9 in SNE and BS15 groups were significantly higher than those in the control group (*P* < 0.05). **(E)** mRNA expression levels of Cytc *c*, Apaf-1, and Caspase-9 in the SNE group were also significantly higher than that of BS15 group but to a limited extent (*P* > 0.05).

Figure 9 presents results on apoptosis-related proteins in the ileum. Although no significant change in Bcl-2 protein content was detected (*P* > 0.05), mRNA expression levels of Bcl-2 in the SNE and BS15 groups were significantly lower than those in the control group (*P* < 0.05). Neither protein content nor mRNA expression level of Bax showed significant changes among the three groups (*P* > 0.05). Both mRNA expression level and protein content of Caspase-3 and Bax/Bcl-2 ratio in the BS15 group were lower than those in the SNE group (*P* < 0.05) and higher than those in the control group (*P* < 0.05). mRNA expression levels of Cytc *c*, Apaf-1, and Caspase-9 in SNE and BS15 groups were significantly higher than those in the control group (*P* < 0.05). mRNA expression levels of Cytc *c*, Apaf-1, and Caspase-9 in the SNE group were also significantly higher than those of the BS15 group but to a limited extent (*P* > 0.05).

### Cytokines, MMP-2, and Nrf-2

**Figure 10:**
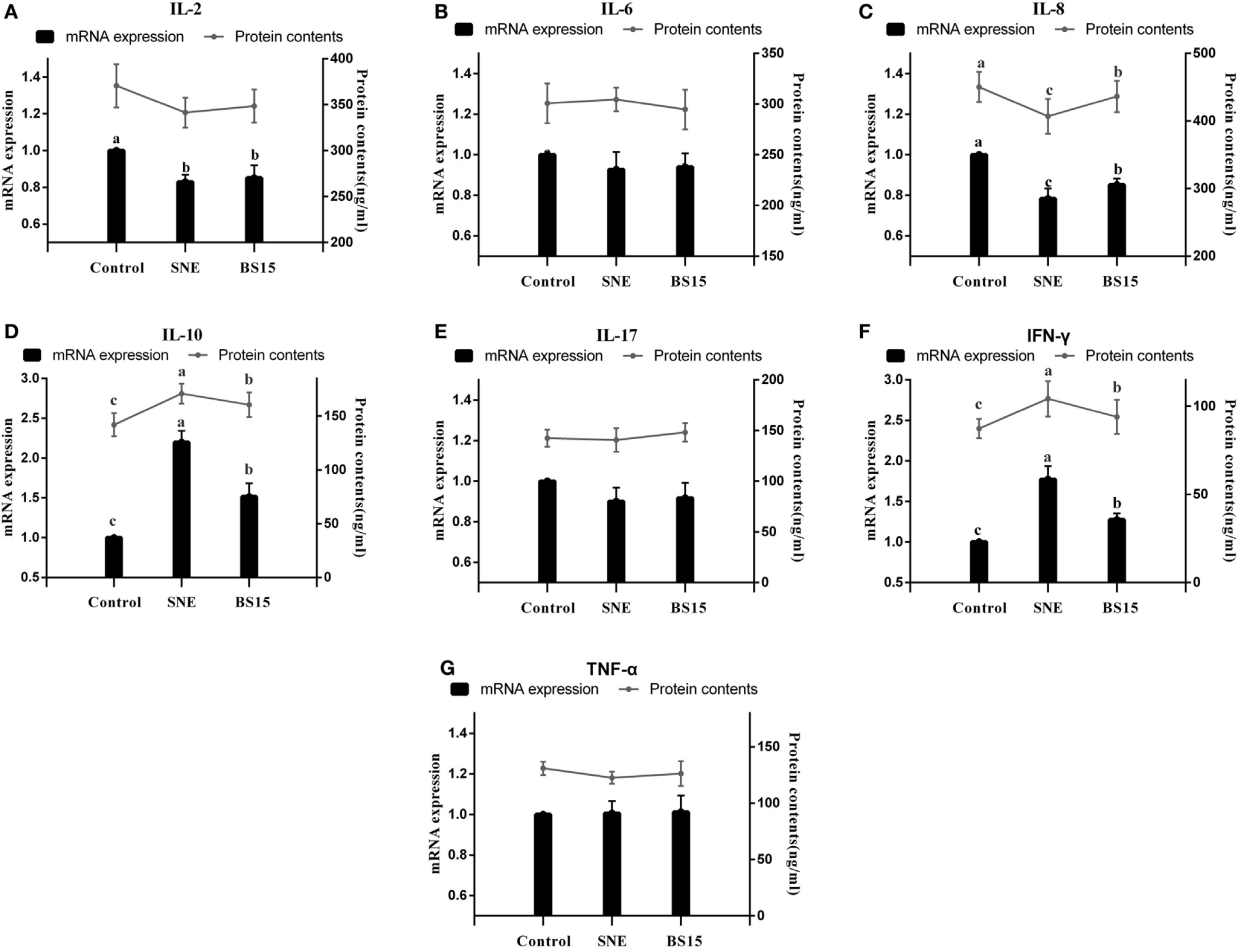
Cytokine levels in the ileum. Bars with different letters significantly differ on the basis of Duncan’s multiple range tests (*P* < 0.05). Data are presented as mean ± SD (six replicates of one chick per cage). TNF-α, tumor necrosis factor-alpha; IFN-γ, interferon-gamma. **(A)** mRNA expression levels of IL-2 were significantly lower in SNE and BS15 groups than in the control group (*P* < 0.05), but no difference in protein contents of IL-2 was observed (*P* > 0.05). **(C)** Both mRNA expression levels and protein contents of IL-8 in the BS15 group were higher than those in the SNE group (*P* < 0.05) but lower than those in the control group (*P* < 0.05). **(D,F)** In the SNE group, both mRNA expression levels and protein contents of IFN-γ and IL-10 were higher than those in the control group (*P* < 0.05). **(D,F)** mRNA expression levels of IFN-γ and IL-10 in the BS15 group were lower than those in the SNE group (*P* < 0.05) but higher than those in the control group (*P* < 0.05). **(B,E,F)** No significant change was detected among levels of other determined cytokines (*P* > 0.05).

**Figure 11:**
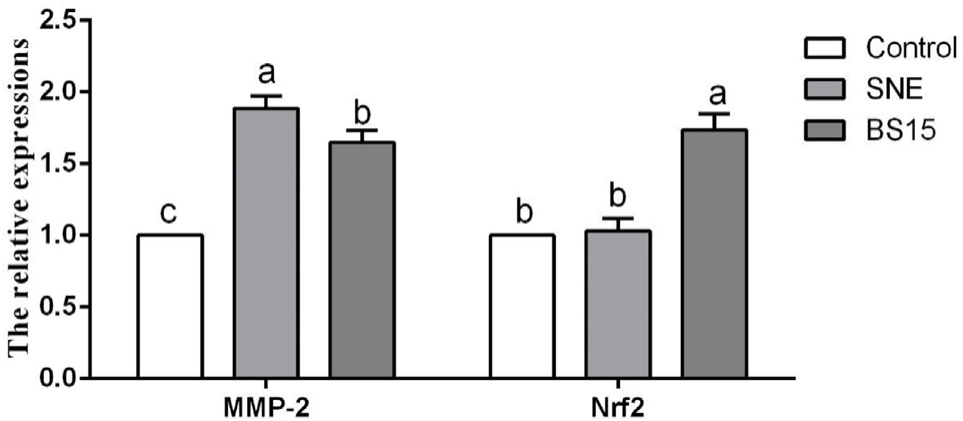
Levels of MMP-2 and Nrf-2 in the ileum. Bars with different letters significantly differ on the basis of Duncan’s multiple range tests (*P* < 0.05). Data are presented as mean ± SD (six replicates of one chick per cage). MMP-2, matrix metalloproteinase-2; SNE, subclinical necrotic enteritis. (1) mRNA expression level of MMP-2 in BS15 group was lower than that in SNE group (*P* < 0.05) but significantly higher than that in the control group (*P* < 0.05). (2) Nrf-2 mRNA expression level was higher in the BS15 group than that in the other two groups (*P* < 0.05).

Figure 10 displays results on cytokine levels in the ileum. mRNA expression levels of IL-2 were significantly lower in the SNE and BS15 groups than those in the control group (*P* < 0.05). However, protein contents of IL-2 showed no difference (*P* > 0.05). Both mRNA expression levels and protein contents of IL-8 in the BS15 group were higher than those in the SNE group (*P* < 0.05) but lower than those in the control group (*P* < 0.05). In the SNE group, both mRNA expression levels and protein contents of IFN-γ and IL-10 were higher than those in the control group (*P* < 0.05). Meanwhile, mRNA expression levels of IFN-γ and IL-10 in the BS15 group were lower than those in the SNE group (*P* < 0.05) but higher than those in the control group (*P* < 0.05). No significant changes were observed in the levels of other determined cytokines (*P* > 0.05). As shown in Figure 11, mRNA expression level of MMP-2 in the BS15 group was lower than that in the SNE group (*P* < 0.05) but significantly higher than that in the control group (*P* < 0.05). Nrf-2 mRNA expression level was higher in the BS15 group than that in the other two groups (*P* < 0.05).

## Discussion

In animal industry, probiotics are proven to exert beneficial effects on health of animals and to prevent various gut diseases. For example, the dietary probiotic *Bacillus coagulans* can promote growth performance and improve meat quality of Guangxi Yellow chicken (24). Pan et al. (25) provided evidence that probiotic supplementation can protect weaned pigs against enterotoxigenic *Escherichia coli* K88 challenge by enhancing immune responses and attenuating intestinal damage. To further gain insights into the direct and indirect effects of probiotic strains on hosts and the mechanisms underlying such effects, numerous studies have been conducted from various aspects. Guo et al. (26) observed that supplementation of the probiotic *Bacillus subtilis* for rabbits can improve growth performance, intestinal homeostasis, immune organ index, innate immune response, and disease resistance. They also discovered that induction of β-defensin may be related to the beneficial effects of *B. subtilis*. In addition, Eeckhaut et al. (8) reported that probiotic *Butyricicoccus pullicaecorum* can reduce the abundance of some potentially important pathogens in the caeca and ileum and thus prevent NE in broilers. In our recent study on *Lactobacillus plantarum*, the giant panda-isolated probiotic can protect mice from enterotoxigenic *E. coli* infection by attenuating inflammation and maintaining integrity of the intestinal epithelial barrier (27). However, although the assumption that probiotics can protect the body from gut diseases by enhancing gut immunity (28), improving gut development (29), and altering gut microbiota (30) has been well accepted, the exact mechanisms on how beneficial microbes cooperate with immunity to provide resistance to pathogens and the role of probiotics in immunity remain unclear (31, 32).

Feng et al. (33) demonstrated in a previous study that NE changed composition of ileal bacterial microbiota of broiler chickens by significantly reducing the abundances of *Lactobacilli* and *Lactobacillus aviaries;* this result correlated negatively with the lesions of NE. As we have discovered in our recent study, *L. johnsonii* BS15 may enhance intestinal development and balance microbiota in jejunum and ileum and thus control growth performance in broilers with SNE (19). Hence, based on our previous results, the present study aimed to prove the effect of BS15 on gut immunity in preventing SNE in broilers. Aside from the ileum, the cecal tonsil—one of the intestinal sections affected by lesions—was also selected and partially examined. Cecal tonsil is the largest lymphoid organ of avian gut-associated lymphoid tissues, from which both T and B cells are present in the germinal centers (34). Cecal tonsil is also an important component of the mucosal immune system and performs unique immune functions (35). However, in our present study, no changes were determined in this organ.

Subclinical NE is characterized by poor growth performance without mortality. Previous statistical analyses showed strong correlations between SNE and increased FCR and retarded growth rate of broilers (36). Poor growth performance is considered to be primarily caused by coccidia-induced leakage of proteins, including plasma, into the lumen of small intestines; this condition provides nutrient substrates for rapid replication of CP and damages digestive ability of the small intestines (37). According to the present results on growth performance, SNE exerted a significant negative influence on FCR, agreeing with our recent results (5) and those of other former studies (38, 39), whereas BS15 can be effective in controlling growth performance under SNE. Jayaraman et al. (40) also reported that *B. subtilis* PB6 can control growth performance of broilers with SNE. Results also showed that BS15 mainly exerts its beneficial effects by influencing performance at the starter phase given the significantly improved growth performance (higher ADG and lower FCR) against SNE that was observed when adding BS15 before SNE infection, whereas limited effects were observed in the treatment group, in which BS15 was added after the development of SNE. This phenomenon revealed that BS15 may be ineffective when SNE has already developed. These results provide important information on the study and application of BS15. According to histological examinations, SNE significantly damaged villi structure and integrity of lamina propria, showing agreement with recent studies on SNE (41). Nevertheless, broilers in BS15 group were free from damaged villi structure. Considering the important role of the small intestine on absorptive, metabolic, and digestive functions (42), BS15 may have decreased FCR caused by SNE by maintaining integrity of the small intestine.

Avian IgA is present in the majority of intestinal cells, shows similarity to mammalian IgA, and releases sIgA into the gut lumen through transepithelial transport (43). IgA is widely considered critical for protecting mucosal surfaces against toxins, viruses, and bacteria by neutralizing or preventing these pathogens from binding to the mucosal surface. sIgA also plays a role in maintenance of mucosal homeostasis, which determines the composition of intestinal microbiota and influences the development of systemic immunity (44). According to results on Ig detection, the numbers of IgA^+^ B cells and contents of IgA and sIgA were significantly decreased by SNE infection but controlled by BS15. The same trend was also observed on the level of IgG, one of the three major Ig classes in intestinal mucosal immunity of chickens (45). Results indicated that BS15 can enhance Igs to prevent SNE in broilers.

Changes in T cell subsets are considered one of the most important indicators of the level of intestinal mucosal immunity. T cells can be divided into subsets based on the cell surface proteins that they express, specifically CD3, the molecular surface marker of mature T cells. CD4^+^ T cells are associated with major histocompatibility complex (MHC) class II molecules and act as helper or inflammatory T cells in response to exogenous antigens, whereas CD8^+^ T cells are associated with MHC class I molecules and play a crucial role as cytotoxic T cells in response to endogenous antigens (46). CD4^+^/CD8^+^ ratio is a direct index for evaluating the condition of body immunity (47). Recent studies demonstrated that probiotics can improve T cell subsets through regulation of gut microbiota (48). Huang et al. (49) observed that combined probiotics consisting of *Streptococcus faecalis, Clostridium buthricum*, and *Bacillus mesentericus* can induce T cell subsets in the intestine of broiler chicks. These probiotics mainly cause an influx of CD8^+^ T cells into the intestinal mucosa; this influx may enhance intestinal immunity. A previous study on human subjects also showed that dietary supplementation of probiotic *Bacillus polyfermenticus*, which can regulate gut microbiota, possesses a potentially positive effect on immune function by modulating the number of immune cell populations, such as CD4^+^ and CD8^+^ T cells (50). In the present study, significantly negative changes were observed in LPLs of the ileum of the SNE group. Olkowski et al. (3) provided evidence that the pathological process of NE in small intestines spreads first throughout the lamina propria, moves toward the center of villus, and then affects the epithelium, thus agreeing with our results. MMP-2, which plays a key role in mediation of collagen degradation in soft connective tissues, was induced by SNE and can therefore be considered an indicator of the disease (51). In the present study, in the ileum of the SNE group, we also detected significantly high mRNA expression levels of MMP-2, which was also controlled by BS15, suggesting that BS15 may downregulate MMP-2 and thus protect LPL in the ileum from SNE.

Antioxidant enzymes, including CAT, SOD, and GSH-Px, play important roles in degradation of superoxide anions and hydrogen peroxide and protect against oxidative stress (52). In broilers, antioxidant capacity of intestinal mucosa is also crucial for healthy intestinal function. Additionally, high levels of MDA contents imply enhancement of lipid peroxidation and accumulation of lipid peroxides in intestinal mucosa and significant oxidative damage to a wide range of biological molecules, including DNA, lipids, proteins, and carbohydrates (53). In the present study, BS15 exhibited a positive effect on antioxidant ability by significantly increasing T-AOC, SOD, and CAT activities and decreasing MDA level, suggesting the ability of BS15 to improve antioxidant capacity in the small intestine. *Lactobacilli* modulate epithelial cytoprotection through the Nrf-2 pathway, which is closely related to oxidative stress (54). In the present results, mRNA expression level of Nrf-2 in the BS15 group significantly increased, and this event may be correlated with improved antioxidant capacity. Oxidative damage can induce molecular lesions and trigger apoptosis (55). Proteins of Bcl-2 family located on the mitochondrial membrane can alter mitochondrial membrane permeability and trigger apoptosis (56). High Bax/Bcl-2 ratio is related to increased vulnerability to apoptotic activation (57). In the present study, the highest Bax/Bcl-2 ratio was observed in the SNE group, suggesting that mitochondria-mediated apoptosis was activated by SNE. BS15 also effectively controlled the side effects caused by SNE as indicated by the significantly lower Bax/Bcl-2 ratio in the BS15 group than in the SNE group. Once the balance between Bcl-2 and Bax is disrupted, caspase-dependent apoptotic pathway can be activated, resulting in increases in Cytc *c*, Apaf-1, Caspase-3, and Caspase-9. Briefly, Cytc *c* leaks out through the holes formed by Bax in the mitochondrial membrane, triggering the association of Apaf-1 to form an apoptosome; caspase-9 is activated through the apoptosome, which then triggers Caspase-3 activation and ultimately causes cell apoptosis (58). In the present study, all results of apoptosis-related proteins were in correspondence with the caspase-dependent apoptotic pathway, indicating that BS15 may control oxidative damage in the small intestine and regulate intestinal apoptosis while protecting broilers from SNE.

As determinants and modulators of immune pathology, cytokines are present in the normal intestinal mucosal membrane and function in mucosal immunity (59). Under various pathologic circumstances, the intestine has been proposed as a possible source of pro-inflammatory cytokines (60). In the present study, both contents and mRNA expression levels of IL-2, IL-8, IL-10, and IFN-γ were influenced by SNE infection and controlled by BS15. Although numerous cytokines in mammalian systems have been studied widely, little work has been performed on non-mammalian systems to date. Some cytokines exhibit different biological activities compared with those of mammals; gene sequences of proteins also differ substantially from those of other mammalian proteins (61). Similar to mammalian IL-2, chicken IL-2 exhibits potential as an immune activator, and treatment of recombinant chicken IL-2 protein induced peripheral blood lymphocytes to express cell surface IL-2 receptors within 48 h and resulted in increased proportions of peripheral blood CD4^+^ and CD8^+^ T cells (62). Chicken IL-2 was also reported to exhibit T cell proliferative activity (63). Therefore, the decreased levels of IL-2 mRNA expression in SNE group indicated inhibition of activation and proliferation of T cells; this result was in agreement with the negative influences of T cell subsets caused by SNE in the present study. Results of IL-8 agree with those of Xu et al. (64) in their study on control of NE by dietary selenium. Possibly, IL-8 is affected by the activation of Nrf-2/antioxidant response pathway, indicating a possible link between changes in IL-8 and antioxidant capacity in the ileum during SNE (31). Recent studies reported that altered production of either IFN-γ or IL-10 may or may not be considered a common marker that is associated with protective immunity against NE (65); increases in IFN-γ (66) and IL-10 (67) were also discussed in research on NE. In our present study, similar changes in both IFN-γ and IL-10 levels were observed in the ileum, suggesting the preventive ability of BS15 supplementation against SNE in broilers. As chicken cytokines remain poorly understood, especially in those the intestines, additional research are needed to demonstrate specific cytokine functions and the relationship between all cytokines and the avian immune system (68).

## Conclusion

Feed supplementation with *L. johnsonii* BS15 may prevent SNE-caused decrease in growth performance of broilers. Changes influenced by BS15 may be related to the enhancement of intestinal immunity of the small intestine. Additional intensive research on improved levels of Igs, better T cell subsets, enhanced antioxidant and anti-apoptotic capacity, and improved cytokines are required before the mechanism of BS15 in prevention of SNE can be determined.

## Ethics Statement

All animal experiments were performed in accordance with guidelines for the care and use of laboratory animals and approved by the Institutional Animal Care and Use Committee of Sichuan Agricultural University (approval number: SYXKchuan2014-187).

## Author Contributions

HW, DZ, and XN designed the experiments. LL, KP, XQ, JL, and BJ performed the experiments. LL, GL, and AK analyzed the experimental data. HW and DZ wrote this paper. All authors read and approved the final manuscript.

## Conflict of Interest Statement

The authors declare that the research was conducted in the absence of any commercial or financial relationships that could be construed as a potential conflict of interest.
